# Long-term retention on antiretroviral therapy among infants, children, adolescents and adults in Malawi: A cohort study

**DOI:** 10.1371/journal.pone.0224837

**Published:** 2019-11-14

**Authors:** Catrina Mugglin, Andreas D. Haas, Joep J. van Oosterhout, Malango Msukwa, Lyson Tenthani, Janne Estill, Matthias Egger, Olivia Keiser

**Affiliations:** 1 Institute of Social and Preventive Medicine, University of Bern, Bern, Switzerland; 2 Dignitas International, Zomba, Malawi; 3 Department of Medicine, College of Medicine, University of Malawi, Blantyre, Malawi; 4 Baobab Health Trust, Lilongwe, Malawi; 5 Institute of Global Health, University of Geneva, Geneva, Switzerland; 6 I-TECH Malawi, Lilongwe, Malawi; University of the Witwatersrand, SOUTH AFRICA

## Abstract

**Objectives:**

We examine long-term retention of adults, adolescents and children on antiretroviral therapy under different HIV treatment guidelines in Malawi.

**Design:**

Prospective cohort study.

**Setting and participants:**

Adults and children starting ART between 2005 and 2015 in 21 health facilities in southern Malawi.

**Methods:**

We used survival analysis to assess retention at clinic level, Cox regression to examine risk factors for loss to follow up, and competing risk analysis to assess long-term outcomes of people on antiretroviral therapy (ART).

**Results:**

We included 132,274 individuals in our analysis, totalling 270,256 person years of follow up (PYFU; median per patient 1.3, interquartile range (IQR) 0.26–3.1), 62% were female and the median age was 32 years. Retention on ART was lower in the first year on ART compared to subsequent years for all guideline periods and age groups. Infants (0–3 years), adolescents and young adults (15–24 years) were at highest risk of LTFU. Comparing the different calendar periods of ART initiation we found that retention improved initially, but remained stable thereafter.

**Conclusion:**

Even though the number of patients and the burden on health care system increased substantially during the study period of rapid ART expansion, retention on ART improved in the early years of ART provision, but gains in retention were not maintained over 5 years on ART. Reducing high attrition in the first year of ART should remain a priority for ART programs, and so should addressing poor retention among adolescents, young adults and men.

## Introduction

In 2014 UNAIDS released the “90-90-90” targets and antiretroviral therapy (ART) for prevention of HIV transmission became key to the response to the global HIV epidemic [1]. With the “90-90-90” targets UNAIDS aims to end the HIV epidemic by ensuring that the majority of people infected with HIV are on effective ART and can no longer transmit the virus: by 2020, 90% of all people living with HIV should know their status, 90% of people diagnosed should receive ART, and 90% of the people on ART should achieve viral load suppression.

The latest World Health Organization (WHO) recommendation that all people living with HIV should initiate ART regardless of clinical or immunological stage facilitates early uptake of ART, but long-term retention on ART is crucial for the success of the 90-90-90 strategy, as people who stop treatment experience HIV replication and may acquire ART resistance and transmit the virus [2,3]. Therefore, treatment programs face the challenge to extend ART to all HIV-infected patients, and at the same time retain the expanding number of patients on lifelong ART [4,5]. Studies suggest that people who initiate ART in a less advanced stage of the disease have worse retention than those who are sick when they begin therapy [6–8]. High rates of ART uptake among asymptomatic PLHIV due to universal ART eligibility may thus lead to worse retention [9].

Several systematic reviews have shown that retention on ART is suboptimal and treatment programs need to implement interventions to improve retention to meet UNAIDS targets [10–12]. Retention in care is particularly challenging among adolescents [13,14], pregnant women, and their HIV-exposed children [7,15] and the latest WHO guidelines promote differentiated care models to address the different needs for HIV care services for diffent patient populations. Intervention to improve retention that are targeted to specific populations including teen friendly services [16] or fewer clinic visits for patients stable on ART [16] are promising interventions. Several studies have shown that loss to follow-up is a substantial problem in Malawi’s ART programme, reported attrition is highest in adolescents [13,14] and women who started ART in the Option B+ programme [7,8]. Most studies have analyzed loss to follow-up in either adults or children and in limited geographical areas.

We examine retention in care by year of starting ART to assess the impact of expanded access to ART on retention. We included patients from a large part of Malawi and from all age groups to identify populations in the greatest need for tailored interventions to improve retention.

## Methods

### The Malawi ART programme

Malawi introduced free ART in 2004, using a public health approach that standardizes ART regimens and clinically monitors patients for toxicity and treatment failure, in 2011 viral load (VL) monitoring was introduced. Patients are followed monthly for the first 6 months, and every two or three months thereafter. In 2003, Malawi issued the first national HIV management guidelines. Recommendations for when to start ART, and which ART regimens should be used, have changed over time (S1 Table), generally in line with WHO guidance. National guidelines were first revised in April 2006 (when the CD4 threshold for ART eligibility changed from 200 to 250 cells/μl), and again in April 2008 (CD4 thershold remained at 250 cells/μl), July 2011 (CD4 threshold changed to 350cells/μl and all pregnant and breastfeeding women became eligible for lifelong ART when Option B+ was introduced; additionally lifelong ART for all children under 2 years was introduced), and in July 2014 (CD4 threshold changed to 500cells/μl) [17]. In May 2016 new guidelines were published, introducing universal test-and-treat for all persons living with HIV. Between 2005 and 2011, large ART clinics started using an electronic medical record system (EMRS) operated by the Baobab Health Trust (www.baobabhealth.org) [18]. Recorded characteristics in the EMRS system at ART initiation include sex, age, reason for starting ART (WHO clinical stage, CD4 cell count, pregnant or breastfeeding women, age below 5 years). Registration and follow-up data for patients starting ART are routinely collected. To minimize the risk of incorrectly documenting drug dispensation and visits, healthcare workers used barcode scanners and recorded drug dispensation prospectively at the point of care [18], except during occasional outages of the electronic system, when data were collected on paper forms and entered into the system retrospectively. Tracing of patients lost is performed according to policies published by the Ministry of Health of Malawi. Patients who missed an appointment and did not return to the clinic for more than 60 days were traced by expert clients using phone calls or home visits.

### Inclusion criteria

We used data from 21 facilities with an EMRS in central and southern Malawi, which began initiating patients on ART between 2004 and 2011, depending on the facility. We used data up to database closures, which was between April 2015 and December 2015. We selected these facilities because they were using the Baobab Health Antiretroviral Therapy (BART) EMRS. All treatment-naive children and adults who started ART in this period at any of the included facilities were eligible for inclusion. Patients initiating ART in the 6 months prior to database closure were not included in the analysis since they would not be able to meet the criteria for becoming LTFU (i.e. not returning to the clinic for more than 6 months).

### Definitions and outcomes

We defined ART initiation as the first recorded dispensation of ART drugs, and baseline as the date of ART initiation. ART refers to the use of a triple-drug combination therapy. We defined time periods based on the introduction of new national HIV guidelines: from 1.1.2004–31.3.2006; 1.4.2006–31.3.2008; 1.4.2008–30.6.2011; 1.7.2011–30.06.2014 and 1.7.2014–01.05.2016. We combined the first two guideline periods because relatively few patients were enrolled and eligibility criteria did not change substantially (S1 Table).

We defined retention on ART as being alive and on ART. Patients were classified as not retained on ART on the date they stopped treatment, they were lost to follow up (LTFU), or died. Patients transferring to another clinic were censored at the date of transfer. Patients were classified as having stopped treatment if they were known to be alive, but were no longer on ART. LTFU was defined as not having returned to the clinic for more than six months [19,20]. Once classified as LTFU, patients remain in this state even if they later returned to care to avoid bias caused by transient interruptions [21]. The date of LTFU was defined as the day of the patient’s last visit to the clinic.

We used the STROBE cohort reporting guidelines. [22] (S3 Table).

### Statistical analyses

We used descriptive statistics to examine the characteristics of individuals at the start of ART. We performed survival analyses to describe retention, LTFU, transfer-out, death, and stopping ART. We followed patients from ART initiation until death, transfer out, treatment stop, or censored patients administratively when they stopped being at risk of LTFU (i.e. six months before database closure). First, we plotted crude retention percentages and 95% confidence intervals stratified by age at ART initiation, sex, years on ART, and guideline period. Second, we plotted sub-distribution hazard functions for the cumulative incidence of LTFU, transfer-out, death and stopping ART [23]. We considered death, transfer-out, stopping ART and LTFU as competing events. Third, we used univariable and multivariable Cox proportional hazard models to calculate unadjusted and adjusted hazard ratios (HR) with 95% Confidence Intervals (CI) for factors associated with LTFU. To meet the proportional hazard assumptions, we split follow-up time in three periods (0–1 year, >1–2 years, >2–5 years and 6–8 years on ART) and fitted different Cox models for each period. We adjusted the analyses for age, sex, treatment guideline period at ART initiation and reason for starting ART. We used cluster-based robust standard errors to account for clustering of patients within facilities. Data were analysed with STATA 13.0 (STATA Corporation, College Station, TX).

### Ethical approval

The National Health Sciences Research Committee in Malawi and the Cantonal Ethics Committee of Bern in Switzerland granted ethical approval for the study. Individual informed consent was waived since we analyzed routinely collected data only.

## Results

### Baseline characteristics and study population

Between 2005 and 2015, 132,274 individuals met our inclusion criteria, totalling 270,256 person years of follow up (PYFU; median per patient 1.3, Interquartile range (IQR) 0.26–3.1). We excluded patients with prior ART experience (N = 25,491), those who started ART after analysis closure (N = 4,254) and those with other inconsistencies in data (N = 203). Characteristics of patients excluded from the analysis were similar to those included, except that patients from central hospitals and those who initiated ART in clinical stage III or IV were overrepresented among excluded patients (S2 Table). Median age at ART initiation was 32.5 years (IQR 26.3–39.6); 62% of patients were female. Most patients (77.8%) started ART for their own health, and 16.6% were women starting ART under Option B+. The reason for ART initiation was missing for 5.6% of participants. ART sites included 3 central hospitals, 13 district hospitals, 2 health centres, and 3 faith-based hospitals. The number of sites increased from one site in 2005, to 21 in 2015. The number of included patients per site varied from 688 to 25,809.

Median age at ART initiation decreased from 33.8 years in the period 2005–2008, to 31.8 during 2014–2015 (Table 1). The proportion of females who started ART increased from 57.9% to 62.5%, peaking at 65.3% in 2011–2014, after Option B+ was introduced (Table 1). After this point, approximately 25% of patients started ART under Option B+.

**Table 1:** Patient characteristics at initation of antiretroviral therapy (ART), stratified by Malawi national guideline period for ART initiation (2005–2015).

	Guideline Period of initiating ART									
	2005–2008		2008–2011		2011–2014		2014–2015		TOTAL	
Number of patients (%)	13,011	(9·8%)	37,445	(28·3%)	67,887	(51·3%)	13,931	(10·5%)	132,274	(100·0%)
Male	5,474	(42·1%)	15,473	(41·3%)	23,558	(34·7%)	5,224	(37·5%)	49,729	(37·6%)
Female	7,537	(57·9%)	21,972	(58·7%)	44,329	(65·3%)	8,707	(62·5%)	82,545	(62·4%)
Age at ART start (years) (%)										
0–3	333	(2·6%)	1,394	(3·7%)	2,420	(3·6%)	455	(3·3%)	4,602	(3·5%)
4–6	260	(2·0%)	633	(1·7%)	795	(1·2%)	181	(1·3%)	1,869	(1·4%)
7–14	656	(5·0%)	1,378	(3·7%)	2,057	(3·0%)	424	(3·0%)	4,515	(3·4%)
15–24	1,040	(8·0%)	3,219	(8·6%)	10,336	(15·2%)	2,316	(16·6%)	16,911	(12·8%)
25–34	4,803	(36·9%)	14,142	(37·8%)	26,978	(39·7%)	5,384	(38·6%)	51,307	(38·8%)
35–44	3,824	(29·4%)	10,550	(28·2%)	16,664	(24·5%)	3,460	(24·8%)	34,498	(26·1%)
45–54	1,560	(12·0%)	4,311	(11·5%)	5,918	(8·7%)	1,211	(8·7%)	13,000	(9·8%)
55+	535	(4·1%)	1,818	(4·9%)	2,719	(4·0%)	500	(3·6%)	5,572	(4·2%)
Median (IQR)	33.82	(27.65–41.1)	33.72	(27.56–40.7)	31.78	(25.61–38.84)	31.83	(25.18–38.78)	32.49	(26.27–39.64)
Reason for starting ART										
WHO stage III/IV	10,046	(77·2%)	22,562	(60·3%)	26,260	(38·7%)	3,945	(28·3%)	62,813	(47·5%)
CD4 cell count measurement	2,737	(21·0%)	14,043	(37·5%)	20,868	(30·7%)	849	(6·1%)	38,497	(29·1%)
Breastfeeding, pregnancy or Option B+	0	(0·0%)	26	(0·1%)	18,448	(27·2%)	3,433	(24·6%)	21,907	(16·6%)
Pediatric*	33	(0·3%)	313	(0·8%)	1,017	(1·5%)	275	(2·0%)	1,638	(1·2%)
Unknown	195	(1·5%)	501	(1·3%)	1,294	(1·9%)	5,429	(39·0%)	7,419	(5·6%)
Health care level										
Central Hospital	9,722	(74·7%)	13,398	(35·8%)	12,621	(18·6%)	2,821	(20·2%)	38,562	(29·2%)
Distrtrict Hospital	3,289	(25·3%)	22,962	(61·3%)	44,179	(65·1%)	9,225	(66·2%)	79,655	(60·2%)
Health Center	0	(0·0%)	232	(0·6%)	5,954	(8·8%)	1,241	(8·9%)	7,427	(5·6%)
Mission Hospital	0	(0·0%)	853	(2·3%)	5,133	(7·6%)	644	(4·6%)	6,630	(5·0%)

* Pediatric includes starting due to known HIV infection and pediatric WHO stage. Children can also start due to WHO stage III / V and CD4 cell count measurement

### Retention on ART

By the end of the study period, 46% (60,582) of patients were retained on ART at the clinic where they initated treatment, 16% (20,868) had transferred elsewhere, 4.6% (6,040) had died, 33.1% (43,766) were LTFU, and 0.7% (1,018) had stopped ART.

**Fig 1:**
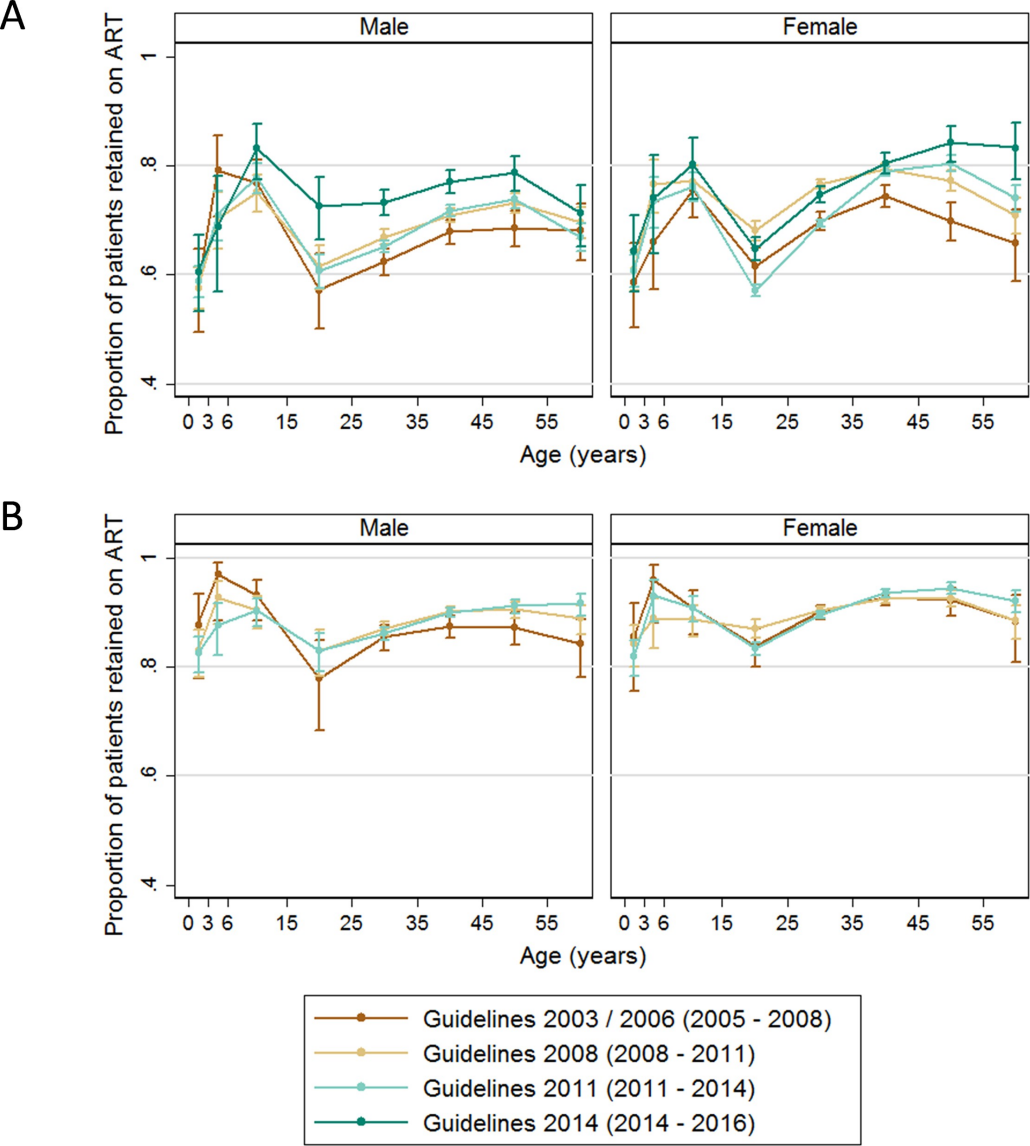
Crude retention at clinic according to gender and guideline periods. (A) at 1 year (B) at 2 years for those who were retained by the end of year 1 C) at 5 years for those who were retained at the end of year 2 note that the scale of the y axis does not start at 0.

Fig 1 shows crude retention on ART for different age groups and guideline periods in the first (A), second (B) and fifth (C) year on ART. Retention on ART differed between age groups and between guideline periods. In the first year on ART, retention improved from the first to the second guideline period for males and females. For male participants retention improved thereafter, with the highest retention observed in the most recent time period. Whereas for female participants this trend was only visible for the older participants (age goups 45–54 and >55 years), for those aged 15–24 retention decreased with the introduction of Option B+ in 2011. Retention in adolescents was lower than in other age groups, for both males and females. Among those retainted on ART for the first year, retention in the second year on ART was similar between guideline periods, and those aged 15–24 had lower retention than other age groups. Retention during the first year of ART was lower than in the second year of ART for all age groups and guideline periods. For those retained on ART by the end of year 2, retention in year 3–5 on ART were lower for men than for women. The drop in retention for those aged 15–24 nearly disappeared for female participants but remained prominent for males (Fig 1).

### Risk factors for LTFU

Table 2 shows the risk of LTFU for different patient groups, stratified by duration on ART. There was no difference between males and females in the univariable model for the first and second year on ART. During years 2–5 on ART, females had a lower risk of becoming LTFU than males (HR 0.85, 95% CI 0.78–0.93) and this difference persisted during the years 6–8. After adjusting for guideline period, age at ART start and reason for starting ART, females had a lower risk of LTFU in all periods after ART initiation compared to males (aHR 1^st^ year 0.75, 95% CI 0.72–0.78; 2^nd^ year 0.73, 95% CI 0.68–0.79; 2–5 years 0.77, 95% CI 0.70–0.84, 6–8 years 0.62 95% CI 0.55–0.69) (Table 2). The risk of LTFU decreased from the first guideline period to the second for the first year of ART (HR 0.81, 95% CI 0.67–0.98), this difference persisted in the multivariable model (aHR 0.80, 0.66–0.97). There were only small differences in LTFU risk between guideline periods for all years on ART (Table 2). Infants aged 0–2 years and young adults aged 15–24 years had the highest risk of LTFU during the years 1–5 on ART in both the univariable and the multivariable model. During the years 6–8 children aged 6–14 had the highest risk of LTFU. Participants initiating ART when they were 35 years or older, had a lower risk of LTFU compared to those aged 25–34 years. In the first year of ART women starting ART due to Option B+ had a higher risk of LTFU compared to those who initiated for their own health (HR 1.90, 95% CI 1.65–2.19; and aHR 1.84,95% CI 1.61–2.11); after one year these differences disappeared.

**Table 2:** Results from Cox Regression Model, Hazard ratios (HR) and adjusted Hazard ratios (aHR) for loss to follow up (LTFU) are shown stratified by years of retention on antiretroviral therapy (ART).

	1^st^ year on ART		2^nd^ year on ART		2–5 years on ART		6–8 years on ART	
	Univariable (N = 132,274), HR (95% CI)	Mulitvariable* (N = 132,274), aHR (95% CI)	Univariable (N = 132,274), HR (95% CI)	Mulitvariable* (N = 132,274), aHR (95% CI)	Univariable (N = 132,274), HR (95% CI)	Mulitvariable* (N = 132,274), aHR (95% CI)	Univariable (N = 132,274), HR (95% CI)	Mulitvariable* (N = 132,274), aHR (95% CI)
**Sex**								
**Male**	1.00 (ref)	1.00 (ref)	1.00 (ref)	1.00 (ref)	1.00 (ref)	1.00 (ref)	1.00 (ref)	1.00 (ref)
**Female**	1.01 (0.95–1.08)	0.75 (0.72–0.78)	0.91 (0.79–1.04)	0.73 (0.68–0.79)	0.85 (0.78–0.93)	0.77 (0.70–0.84)	0.66 (0.60–0.73)	0.62 (0.55–0.69)
**HIV guideline period**								
**Guidelines 2003 / 2006** ^**#**^	1.00 (ref)	1.00 (ref)	1.00 (ref)	1.00 (ref)	1.00 (ref)	1.00 (ref)	1.00 (ref)	1.00 (ref)
**Guidelines 2008**^**†**^	0.81 (0.67–0.98)	0.80 (0.66–0.97)	0.98 (0.79–1.21)	0.96 (0.79–1.18)	1.38 (0.98–1.94)	1.36 (0.97–1.91)	0.97 (0.73–1.30)	0.98 (0.74–1.30)
**Guidelines 2011**^**‡**^	1.08 (0.93–1.25)	0.87 (0.75–1.01)	1.08 (0.84–1.39)	0.96 (0.77–1.21)	1.21 (0.77–1.91)	1.17 (0.76–1.82)	NA	NA
**Guidelines 2014** **	1.02 (0.88–1.18)	0.94 (0.84–1.07)	NA	NA	NA	NA	NA	NA
**Age at ART start (years)**								
**0–2**	1.44 (1.17–1.77)	1.55 (1.27–1.89)	1.49 (1.26–1.78)	1.48 (1.28–1.70)	1.14 (0.96–1.36)	1.06 (0.89–1.27)	1.07 (0.78–1.47)	0.98 (0.71–1.36)
**3–5**	0.91 (0.80–1.04)	0.98 (0.86–1.12)	0.75 (0.54–1.04)	0.72 (0.51–1.02)	1.02 (0.77–1.36)	0.97 (0.74–1.29)	1.03 (0.55–1.94)	0.88 (0.44–1.76)
**6–14**	0.73 (0.66–0.81)	0.78 (0.71–0.85)	0.83 (0.68–1.01)	0.82 (0.65–1.05)	1.06 (0.94–1.21)	1.03 (0.89–1.20)	1.66 (1.47–1.87)	1.48 (1.31–1.66)
**15–24**	1.51 (1.43–1.58)	1.35 (1.29–1.40)	1.52 (1.41–1.63)	1.51 (1.38–1.66)	1.38 (1.26–1.50)	1.42 (1.32–1.53)	1.17 (0.94–1.47)	1.29 (1.05–1.57)
**25–34**	1.00 (ref)	1.00 (ref)	1.00 (ref)	1.00 (ref)	1.00 (ref)	1.00 (ref)	1.00 (ref)	1.00 (ref)
**35–44**	0.74 (0.71–0.77)	0.79 (0.77–0.81)	0.72 (0.64–0.80)	0.70 (0.65–0.76)	0.79 (0.75–0.83)	0.76 (0.72–0.80)	0.70 (0.59–0.82)	0.64 (0.56–0.73)
**45–54**	0.70 (0.66–0.75)	0.77 (0.74–0.80)	0.64 (0.59–0.70)	0.62 (0.55–0.69)	0.69 (0.63–0.75)	0.64 (0.59–0.70)	0.71 (0.60–0.83)	0.63 (0.55–0.73)
**>54**	0.84 (0.74–0.95)	0.92 (0.85–0.99)	0.77 (0.66–0.91)	0.75 (0.64–0.88)	0.82 (0.71–0.95)	0.77 (0.65–0.91)	1.24 (0.97–1.58)	1.09 (0.84–1.40)
**Reason for starting ART**								
**Own health**	1.00 (ref)	1.00 (ref)	1.00 (ref)	1.00 (ref)	1.00 (ref)	1.00 (ref)	1.00 (ref)	1.00 (ref)
**Option B+**	1.90 (1.65–2.19)	1.84 (1.61–2.11)	1.45 (1.08–1.96)	1.34 (0.99–1.82)	1.08 (0.87–1.33)	1.06 (0.97–1.15)	NA	NA

* all models accounted for site heterogeneity using cluster based robus standard errors

^**#**^ (calendar years 2005–2008)

**†** (calendar years 2008–2011)

**‡** (calendar years 2011–2014)

** (calendar years 2014–2016

### Competing risks analysis

**Fig 2:**
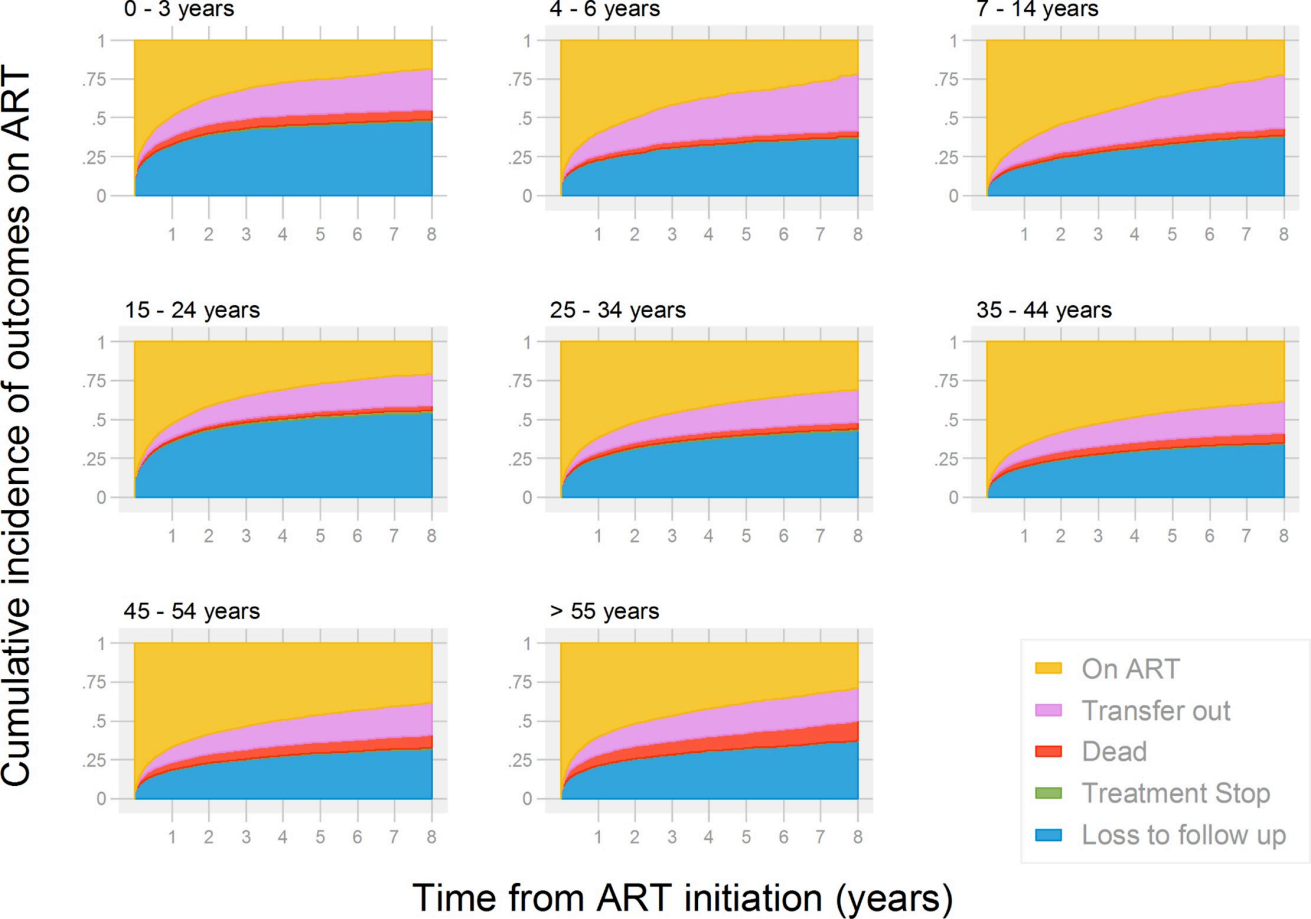
Cumulative incidence of antiretroviral therapy (ART) outcomes for patients at 21 facilities (estimates from competing risk analysis). Treatment outcomes are compared between age groups according to age at ART initiation.

Overall retention on ART was 70.0% at 12 months after ART initiation, 65.2% after 2 years, and 50.7% after 5 years. **Fig 2** shows the competing risks analysis. Most patients who defaulted from care were LTFU in the first year. In the competing risk analysis death was more common among the oldest age group (>55 years of age) and children compared to those aged 25–34, while LTFU was more common among adolescents and young adults.

## Discussion

We compared retention on ART for a large group of patients over a period of 10 years of ART provision in central and southern Malawi. Even though the number of patients and the burden on the health care system increased substantially during the study period of rapid ART expansion, we found that retention on ART improved in the early years of ART, and stabilized thereafter. Retention on ART at the clinic varied between different age groups, and was lowest among infants and young adults aged 15–24. Males were at higher risk of LTFU than females throughout the study period, despite the introduction of Option B+ in 2011, which increased the number of females starting ART. Older PLHIV (>55 years) were more likely to die than younger persons, irrespective of duration on ART.

Strengths of this study are the large sample size, and the ability to compare data over a long time period of HIV treatment and guideline changes during strong program expansion. Our study also has several limitations. All clinics that have an EMRS have large patient populations. One previous study found that LTFU was higher in sites that used EMRS than in sites that did not and our results may therefore not be representative for all HIV clinics in Malawi [8]. We probably underestimated retention on ART, since patients who we recorded as LTFU may be in care elsewhere after silent or undocumented transfer [24,25]. A study in Lilongwe, Malawi found that 40% of patients recorded as LTFU, who were alive and successfully traced were in care elsewhere [26]. Underreporting of mortality is very common in ART programs [27] and some patients LTFU had probably died [28]. In Lilongwe, an analysis of a tracing programme for the years 2006 to 2010 revealed that 30% of patients LTFU had died [29]. Death among those LTFU might be higher in earlier time periods, when many started ART with advance HIV, compared to later periods. We do not know why people were LTFU, but tracing studies found that the most frequent reasons for not returning to care were undocumented transfer, stopping ART, and death [24]. Tracing studies among children are scarce, in a systematic review of outcomes of HIV-positive patients LTFU only 4 of 30 studies included children [30]. They found no difference in mortality, undocumented transfers and interruption of ART between children and adults. Since our study relied on routine, operational data collection by caregivers who were often overburdened, some data may be incorrect. The frequency of data errors is probably low, due to regular supervision visits by teams from the Ministry of Health and its HIV care stakeholders, and because of our additional logical checks after data entry. Our dataset only included variables required for national monitoring, so we could not consider socioeconomic, some anthropometric and other variables that are known to be associated with patients’ ART outcomes [31].

Our results confirm and extend those of earlier studies of retention on ART in Malawi and sub-Saharan Africa. Retention in the first year of ART was similar in a study from Malawi covering the time period between 2004 and 2007 [20]. After 12 months of follow up they found that 69.5% of patients were on ART at the same facility. Another study from northern Malawi, covered a comparable timeframe to ours (from 2005–2012) and found similar retention rates in an area of lower HIV prevalence [32]. Studies from Ethiopia, reporting data from 2005–2011 found comparable retention rates [33,34]. A 2015 systematic review of retention on ART between 2008–2013 found that annual attrition decreased after 24 months, and retention was 65%–70% after 3 years on ART [10]. Some studies in sub-Saharan Africa identified the same baseline characteristics that predicted attrition in our analysis (younger age [32] and male gender[35]). Our finding that retention on ART is particularly low in adolescents was also highlighted by other studies from Sub-Saharan Africa [36–39].

Malawi has successfully scaled up ART and sustained initial retention levels in those starting ART more recently. However, there are differences in retention between different patient populations, and patients who are not retained or have inadequate adherence to treatment are at a high risk of viral rebound and drug resistance [40,41]. Programs should consider implementing feasible, evidence-based interventions to promote adherence and retention, including adherence clubs, adherence counseling, text message reminders by telephone, and treatment supporters [42,43].

In conclusion, in central and southern Malawi, retention on ART did not change much over time, even though the number of patients on ART increased rapidly over time and patients starting ART were increasingly asymptomatic and in good health. Since mortality on ART was higher among older participants, it should be further investigated whether mortality in this group is higher compared to HIV negative people of comparable age. Reducing high attrition in the first year of ART should remain a priority for ART programs, and so should addressing poor retention among adolescents, young adults and men.

## Supporting information

S1 Table Malawi national guidelines 2003–2015. (DOCX)

S2 Table Comparison of excluded and included patients. (DOCX)

S3 Table STROBE checklist. (DOCX)
