# Supplementary material for: Long-term retention on antiretroviral therapy among infants, children, adolescents and adults in Malawi: A cohort study
Source: PLoS One. 2019 Nov 14;14(11):e0224837. doi: 10.1371/journal.pone.0224837 (PMC6855432; doi:10.1371/journal.pone.0224837)
Supplement: S1 Table — (DOCX) [file pone.0224837.s001.docx]

S1 Table: Malawi national guidelines 2003 – 2015

| **Edition** | **Year published** | **ART eligibility adults** | | | | **ART eligibility children** | | **First Line Treatments** |
| --- | --- | --- | --- | --- | --- | --- | --- | --- |
|  |  | ***CD4 threshold*** | ***Clinical stage*** | ***TLC*** | ***other*** | ***Clinical stage*** | ***CD4 % / cell count*** |  |
| 1^st^ Guideline Edition | 2003 | < 200/mm3 | WHO stage 3 / 4 | WHO stage 2 and TLC < 1200 |  | <18 months: confirmed HIV +, WHO pediatric stage 3  >18 months: WHO stage III | <18 months: WHO stage 1 or 2 and CD4% < 15%  >18 months: WHO stage 2 and CD4% < 20% | d4T 3TC NVP  (alternative first line regimesn: AZT 3TC NVP, d4T 3TC EFV, AZT ddI NFV) |
| 2^nd^ Guideline Edition | 2006 | < 250/mm3 | WHO stage 3 / 4 | WHO stage 2 and TLC < 1200 |  | < 18 months WHO pediatric clinical stage 4  > 18 months WHO pediatric clinical stage 3 or 4 | > 18 months: CD4% under threshold  WHO stage 2 and TLC < threshold | d4T 3TC NVP  (alternative first line: AZT 3TC NVP, d4T 3TC EFV) |
| 3^rd^ Guideline Edition | 2008 | < 250/mm3 | WHO stage 3 / 4 | WHO stage 2 and TLC < 1200 |  |  |  | d4T 3TC NVP  (alternative first line: AZT 3TC NVP, d4T 3TC EFV) |
| 1^st^ integrated guideline edition | 2011 | < 350/mm3 | WHO stage 3 / 4 |  | Lifelong ART for: Pregnant and breastfeeding women; children under 2 years; children 2‐4 years in WHO stage 1 and 2 with CD4 <750mm3 |  |  | 6 different 1^st^ line regimens, 3 for initiation: d4T, 3TC, NVP; AZT, 3TC, NVP; TDF, 3 |
| 2^nd^ integrated guideline edition | 2014 | ≤ 500/mm3 | WHO stage 3 / 4 | - | Start all: Pregnant or breastfeeding women; Children under 5 years with confirmed HIV infection; | Infant und | CD4 cell count ≤ 500/mm3 | 7 different 1^st^ line regimens, 2 for initiation: TDF, 3TC, NVP or AZT, 3TC, NVP for children |
