# Supplementary material for: Long-term retention on antiretroviral therapy among infants, children, adolescents and adults in Malawi: A cohort study
Source: PLoS One. 2019 Nov 14;14(11):e0224837. doi: 10.1371/journal.pone.0224837 (PMC6855432; doi:10.1371/journal.pone.0224837)
Supplement: S2 Table — (DOCX) [file pone.0224837.s002.docx]

S2 Table: Comparison of excluded and included patients.

| **BASELINE CHARACTERISTIC** | **Included** | | **Excluded** | | | | **Total** | | | |
| --- | --- | --- | --- | --- | --- | --- | --- | --- | --- | --- |
| Number of patients (%) | 132274 | (79,4%) | |  | 34295 | (20,6%) | |  | 166569 | (100,0%) |
| Sex n (%) |  |  | |  |  |  | |  |  |  |
| Male | 49729 | (37.6%) | |  | 13333 | (38.9%) | |  | 63062 | (37.9%) |
| Female | 82545 | (62.4%) | |  | 20962 | (61.1%) | |  | 103507 | (62.1%) |
| Age at ART start (%) |  |  | |  |  |  | |  |  |  |
| 0-3 | 4602 | (3.5%) | |  | 635 | (1.9%) | |  | 5237 | (3.1%) |
| 4-6 | 1869 | (1.4%) | |  | 510 | (1.5%) | |  | 2379 | (1.4%) |
| 7-14 | 4515 | (3.4%) | |  | 1523 | (4.4%) | |  | 6038 | (3.6%) |
| 15-24 | 16911 | (12.8%) | |  | 3714 | (10.8%) | |  | 20625 | (12.4%) |
| 25-34 | 51307 | (38.8%) | |  | 11230 | (32.7%) | |  | 62537 | (37.5%) |
| 35-44 | 34498 | (26.1%) | |  | 8815 | (25.7%) | |  | 43313 | (26.0%) |
| 45-54 | 13000 | (9.8%) | |  | 3424 | (10.0%) | |  | 16424 | (9.9%) |
| 55+ | 5572 | (4.2%) | |  | 1522 | (4.4%) | |  | 7094 | (4.3%) |
| Missing | 0 | (0.0%) | |  | 2922 | (8.5%) | |  | 2922 | (1.8%) |
| Median (IQR) | 32.49 | (26.27-39.64) | |  | 33.46 | (26.84-40.62) | |  | 32.68 | (26.37-39.82) |
| Guidelines 2003 / 2006 (2005 - 2008) | 13011 | (9.8%) | |  | 1483 | (4.3%) | |  | 14494 | (8.7%) |
| Guidelines 2008 (2008 - 2011) | 37445 | (28.3%) | |  | 6408 | (18.7%) | |  | 43853 | (26.3%) |
| Guidelines 2011 (2011 - 2014) | 67887 | (51.3%) | |  | 14100 | (41.1%) | |  | 81987 | (49.2%) |
| Guidelines 2014 (2014 - 2016) | 13931 | (10.5%) | |  | 12304 | (35.9%) | |  | 26235 | (15.8%) |
| Reason for starting ART n (%) |  |  | |  |  |  | |  |  |  |
| WHO stage III/VI adult | 62813 | (47.5%) | |  | 19289 | (56.2%) | |  | 82102 | (49.3%) |
| CD4 cell count measurement | 38497 | (29.1%) | |  | 6453 | (18.8%) | |  | 44950 | (27.0%) |
| Breastfeeding, Pregnancy or Option B+ | 21907 | (16.6%) | |  | 3313 | (9.7%) | |  | 25220 | (15.1%) |
| Pediatric | 1638 | (1.2%) | |  | 305 | (0.9%) | |  | 1943 | (1.2%) |
| Unknown | 7419 | (5.6%) | |  | 4935 | (14.4%) | |  | 12354 | (7.4%) |
| Health care level n (%) |  |  | |  |  |  | |  |  |  |
| Central Hospital | 38562 | (29.2%) | |  | 12212 | (35.6%) | |  | 50774 | (30.5%) |
| Distrtrict Hospital | 79655 | (60.2%) | |  | 17596 | (51.3%) | |  | 97251 | (58.4%) |
| Health Center | 7427 | (5.6%) | |  | 2717 | (7.9%) | |  | 10144 | (6.1%) |
| Mission Hospital | 6630 | (5.0%) | |  | 1770 | (5.2%) | |  | 8400 | (5.0%) |
